# 

**Published:** 1844-11

**Authors:** 


### THE WESTERN JOURNAL OF MEDICINE AND SURGERY:

EDITED BY
DANIEL DRAKE, M. D.
AND
LUNSFORD P. YANDELL, M. D.
PROFESSORS IN THE LOUISVILLE MEDICAL INSTITUTE,
AND
THOMAS W. COLESCOTT, M. D.

NO. LVIX.—NOVEMBER, 1844.

NEW SERIES.
VOL. II.—NO. V.

LOUISVILLE, KY.
PUBLISHED MONTHLY, BY PRENTICE AND WEISSINGER,
PROPRIETORS AND PRINTERS.
1844.

*This Number contains 4 sheets—Postage under 100 miles 6 cents, over 100 miles 10 cents*

